# Feasibility of acromial periosteal flap technique for enhanced reconstruction of acromioclavicular joint dislocations: a cadaveric study

**DOI:** 10.1186/s13018-026-07110-w

**Published:** 2026-07-16

**Authors:** Ole Somberg, Eckart Förster, Thomas Rosteius, Maria Bernstorff, Thomas Schildhauer, Matthias Königshausen

**Affiliations:** 1 Ruhr University Bochum, BG University Hospital Bergmannsheil, Department of General and Trauma Surgery, Buerkle-de-la-Camp-Platz 1, 44789 Bochum, Germany; 2 Ruhr University Bochum, Institute of Anatomy, Universitätsstraße 150, 44801 Bochum, Germany

**Keywords:** Acromioclavicular joint (ACJ), Surgical technique, Periosteal flap, Stability, Cadaveric study

## Abstract

**Aims:**

The aim of this study was to test the feasibility of mobilizing an acromial periosteal flap (APF) and to evaluate its potential to provide locally available, additional biological autologous augmentation for acute and chronic acromioclavicular joint (ACJ) injuries.

**Methods:**

Ten cadaveric upper extremities (5 right, 5 left; 3 female, 7 male) with an average age of 82 ± 3.7 years were used. A direct surgical approach to the ACJ was performed, and the APF was mobilized from three sides of the acromion, leaving the side closest to the clavicle intact. The flap was folded over onto the clavicle and secured with a transosseous suture. Measurements of flap length, width, and clavicular overlap were recorded.

**Results:**

Mobilization of the APF was successfully achieved in all specimens. The flaps appeared clinically stable and tear-resistant, with an average length of 2.3 ± 0.5 cm, width of 1.75 ± 0.5 cm, and clavicular overlap of 1.29 ± 0.6 cm.

**Conclusion:**

Our study demonstrated the anatomical feasibility of preparing an acromial periosteal flap (APF). This anatomical proof of concept may provide the basis for potential future clinical applications as an additional option for biological augmentation in ACJ reconstruction. However, further biomechanical investigations and clinical studies are necessary to evaluate whether this technique can translate into improved stability and functional outcomes.

**Level of evidence:**

V.

## Introduction

Acromioclavicular joint (ACJ) injuries, accounting for approximately 9% of all shoulder girdle injuries, are among the most common injuries in this region. These injuries typically result from direct trauma to the shoulder or a fall onto an outstretched, adducted arm [2, 5]. ACJ dislocations are classified according to the Rockwood classification system into six distinct types [8, 16]. Additionally, they can be categorized as acute or chronic, depending on the time elapsed since the injury [7]. The management of ACJ dislocations varies based on the injury type and whether the presentation is acute or chronic. Treatment strategies may be either conservative or operative, depending on factors such as the severity of the dislocation, patient needs, and functional demands. Previous studies have already demonstrated that both the AC and CC ligaments should be reconstructed to achieve optimal bidirectional stability [1]. The superior acromioclavicular ligament complex (ACLC) is crucial for maintaining horizontal stability in the acromioclavicular joint. Maier et al. identified four distinct types of ACLC injuries and advocated type-specific repair techniques [10]. As shown, the ACLC plays a critical role in ACJ stability. Building on these findings, our study aimed to provide an additional, locally available augmentation through the use of an acromial periosteal flap (APF). In foot surgery, periosteal flaps are also utilized to address ankle instability, offering a viable surgical technique to enhance joint stability [4, 12, 21]. The aim of this study was to test the feasibility of mobilizing an APF and to evaluate its potential to provide locally available, additional biological autologous augmentation for acute and chronic ACJ injuries.

## Methods

### Demographic data

In our cadaveric study, we used ten upper extremities (5 right, 5 left; 3 female, 7 male) with an average age of 82 ± 3.7 years. No pre-existing conditions of the body donors were known. On inspection, there was no evidence of prior surgeries on the affected shoulders. The body donors derive from the donor program of Ruhr-University Bochum. Ethical approval was obtained from the local Ethics Committee (2024-591-f-S).

### Surgical technique

**Fig. 1:**
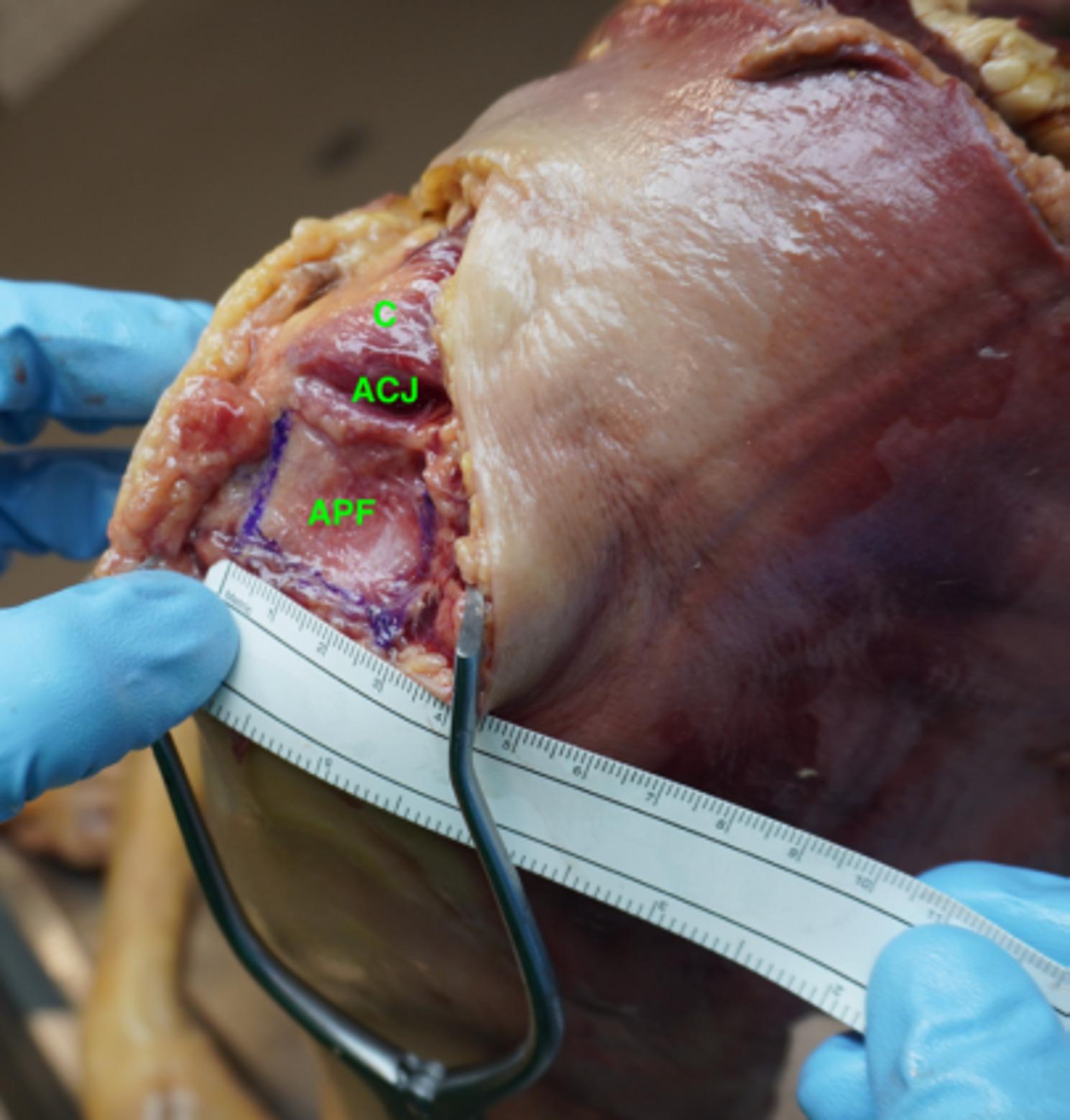
Outline of the acromial periosteal flap (APF), which later will be flipped over the acromioclavicular joint (ACJ) and fixed onto the clavicle (C)

**Fig. 2:**
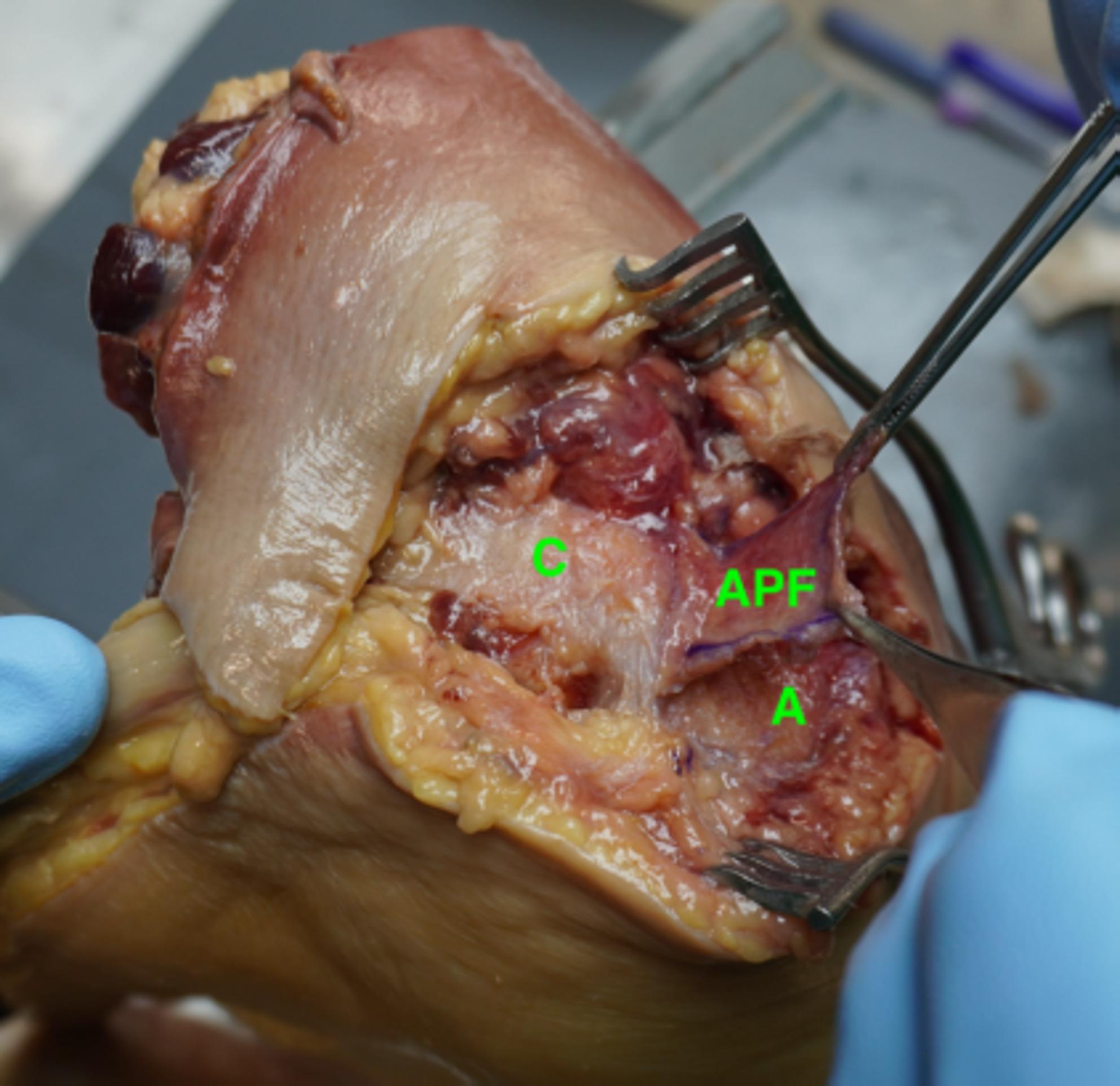
Top-down view after the acromial periosteal flap (APF) was mobilized from three sides of the acromion (A). The side closest to the clavicle (C) remains intact

**Fig. 3:**
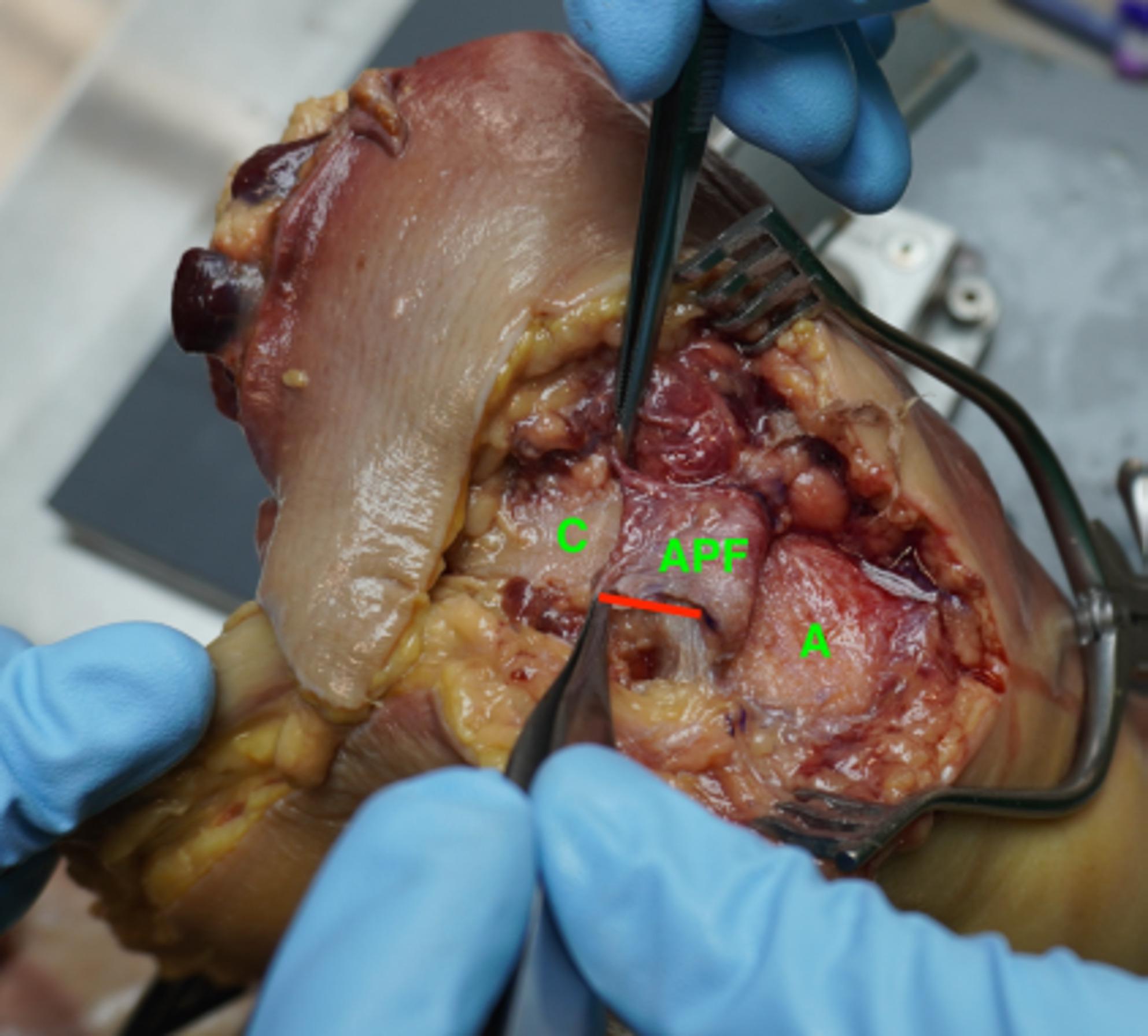
Top-down view after the acromial periosteal flap (APF) was flipped from the acromion (A) onto the clavicle (C). The red line indicates the area of clavicular overlap

**Fig. 4:**
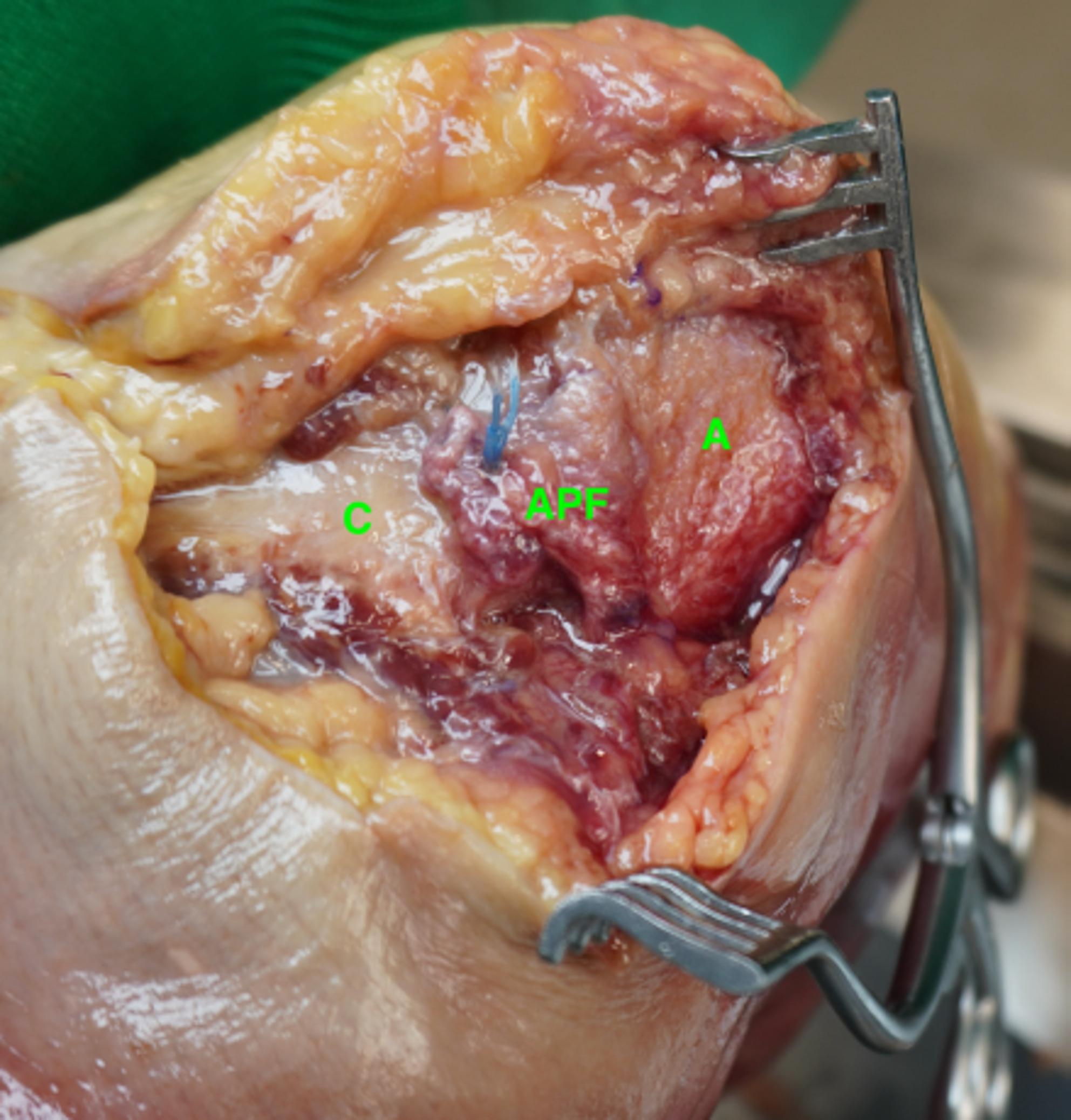
The acromial periosteal flap (APF) flipped from the acromion (A) and fixed to the clavicle (C) using a simple transosseous suture. Reinforcement of the APF with a Krakow suture is also possible

**Fig. 5:**
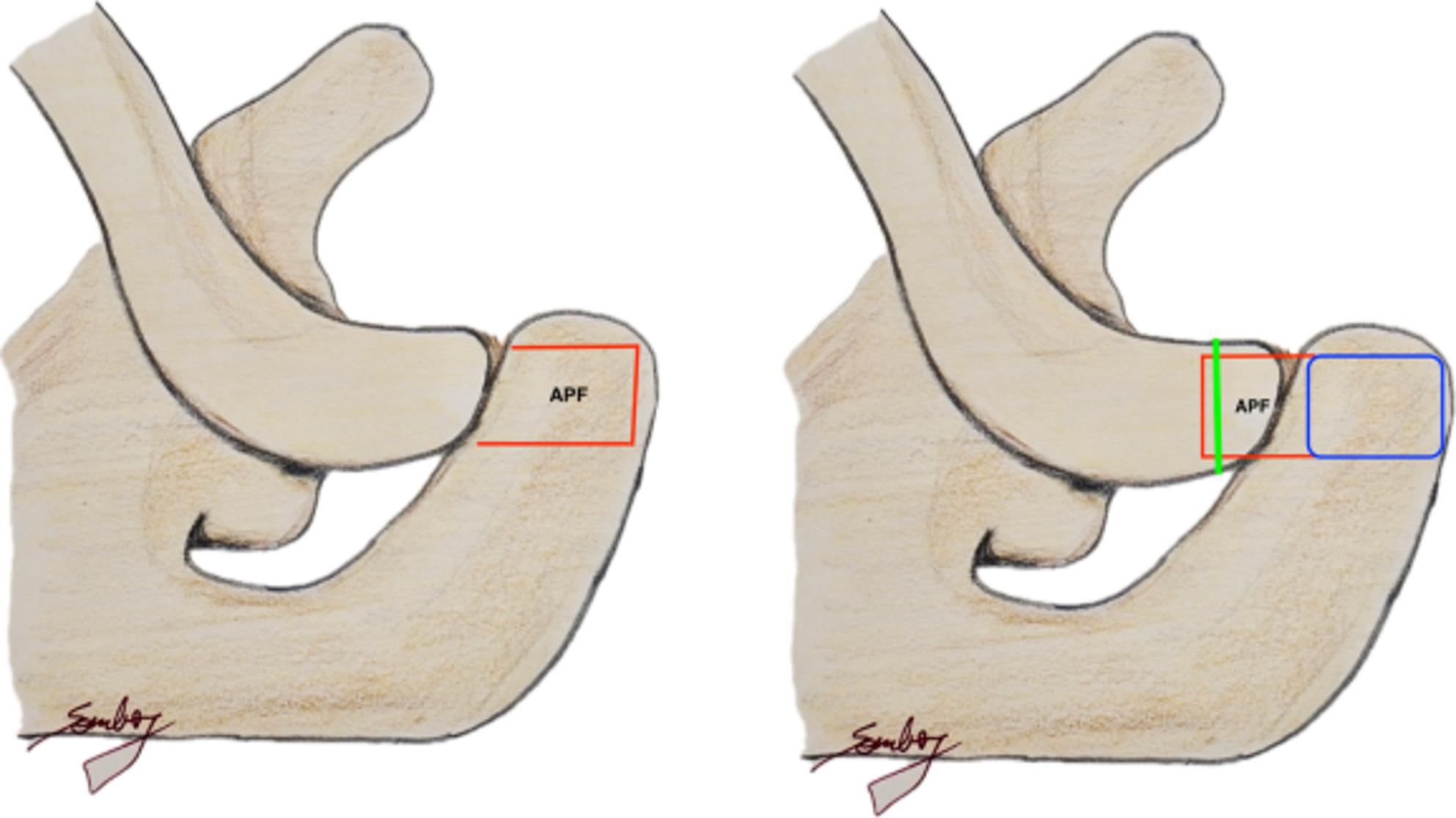
Top view of the ACG. Left side: area from which the APF is mobilized (red). Right side: APF mobilized onto the clavicle (red). Blue indicates the former area of the APF. Green represents the drill hole for passing sutures and fixing the flap

We used a direct approach to the ACJ, with a slightly larger incision to allow for better visualization of the structures. The clavicle, acromion, and ACJ were exposed, and the ACJ capsule was transected. Depending on the size of the acromion, the flap was outlined in such a way that no additional ligament or muscle attachments were disrupted, allowing for the mobilization of a periosteal flap only as shown in Fig. 1. The periosteal flap was elevated from the anterior, posterior, and lateral aspects of the acromion, while its medial border adjacent and parallel to the ACJ was left intact to preserve its attachment. See Fig. 2 for detailed illustration of this step of the procedure. This allowed the flap to remain attached at this site and be folded over onto the clavicle as seen in Fig. 3. To evaluate the acromial periosteal flap, we measured its length, width, and the extent of the clavicular overlap. The clavicular overlap refers to the portion of the flap that, after being folded over, rests on the clavicle and can be secured to it using sutures as part of the stabilization process. An illustration of the final result with a simple transosseous suture can be seen in Fig. 4. Different techniques can be used for securing the flap. In our study, we employed reinforcement using a 2.0 FiberWire (Fa. Arthrex) with a single suture technique or a Krakow suture technique. The sutures were passed through a 2.0 mm drill hole positioned laterally on the clavicle, outside the ACJ articular surface, running from anterior to posterior. The two suture ends were then tied on top of the flap. The Krakow suture allowed the flap to be evenly tensioned and to spread out like a sail, providing a good contact area on the lateral clavicle. A schematic illustration of the surgical technique can be seen in Fig. 5.

### Measurement and statistical analysis

The length and width of the APF were measured using a ruler. Additionally, the extent of bony overlap was determined, representing the portion of the flap that covered the acromion. Mean and standard deviation were calculated for each parameter.

## Results

In all ten cadaveric specimens, the mobilization of the acromial periosteal flap was successfully achieved. Clinically, the flap appeared very stable and tear-resistant. The prepared flaps had an average length of 2.3 ± 0.5 cm, a width of 1.75 ± 0.5 cm, and a clavicular overlap of 1.29 ± 0.6 cm. A clavicular overlap of more than 1 cm seemed sufficient for proper fixation during preparation; however, this assumption will need to be confirmed by future clinical studies.

## Discussion

Over time, therapeutic recommendations and surgical techniques for the treatment of acute and chronic ACJ dislocations have continuously evolved and improved up to the present day. In addition to non-operative treatment, which is used in some cases, current practice in specialized centers favours the TightRope technique—or alternatively, hook plates—for managing acute injuries [3, 20]. While chronic injuries were previously treated using techniques such as the Weaver-Dunn procedure, current management favours the use of the arthroscopically-assisted TightRope technique in combination with tendon grafts [9, 11, 19, 24]. Tendon grafts are necessary because the coracoclavicular and coracoacromial ligaments in chronic cases have lost their healing potential, requiring fresh tendon material to restore stability. Various techniques exist regarding the placement and number of drill channels, as well as whether the tendon graft is passed through the acromion to address horizontal instability [18, 23]. Periosteal flaps are used in foot surgery primarily for reinforcing ligament reconstructions in cases of chronic lateral ankle instability. For example, in the modified Broström-Gould procedure, a periosteal flap is harvested from the distal fibula and used to augment the repair of lateral ankle ligaments, such as the anterior talofibular ligament (ATFL). This technique enhances stability without sacrificing local tendons and provides a scaffold for ligament healing and transformation into functional tissue [4, 12, 21]. Maier et al. identified four distinct injury patterns of the superior acromioclavicular ligament complex (ACLC) in acute ACJ dislocations and emphasized its crucial role in maintaining horizontal stability by preventing clavicular translation. They advocated type-specific repair techniques to optimize stability and functional outcomes [10, 22]. The potential clinical application of the APF may also be supported by experiences from other areas of shoulder surgery. Scheibel et al. reported favourable outcomes after rotator cuff reconstruction augmented with an autologous periosteal flap, with significant improvements in clinical scores and high patient satisfaction [17]. These results highlight that periosteal flaps can be successfully utilized in shoulder reconstruction, underlining the possible future relevance of the APF technique as described in our study. Anatomical studies have described the clavicular footprint of the superior acromioclavicular capsule with a length of approximately 0.43 cm from the lateral end of the clavicle and width of 0.63 cm [14]. The APFs in our study provided a clavicular overlap of 1.29 cm and a width of 1.73 cm, thereby fully covering the superior footprint. Our study has some minor limitations and, as a Level V evidence study, should be regarded as an anatomical feasibility assessment that forms the basis for further biomechanical and clinical research. First, the age of the donors was relatively high, which represents a restrictive aspect of our study, as patients typically affected by ACJ injuries are considerably younger. However, this may also be considered a strength, as it demonstrates that an APF can still be mobilized even in older individuals, despite several studies indicating that the periosteum tends to become thinner and less cellular with increasing age [6, 13, 15]. Second, the majority of donors were male, which may limit the generalizability of the findings. However, APFs were mobilized in both male and female donors. The APF technique offers the potential to provide additional, locally available stability in the treatment of acute cases. In the management of chronic cases, if the graft is not passed through the acromion to address horizontal instability, the flap can serve as an alternative method of biological autologous augmentation. Furthermore, in revision cases of chronic ACJ dislocations—where, for example, the graft is intended to be placed in a figure-of-eight configuration around the clavicle and coracoid, but not additionally used as an AC cerclage because the acromion may have already been drilled and should not be further weakened by another graft passage—this technique may offer a viable alternative.

## Conclusion

Our study demonstrated the anatomical feasibility of preparing an acromial periosteal flap (APF). This anatomical proof of concept may provide the basis for potential future clinical applications as an additional option for biological augmentation in ACJ reconstruction. However, further biomechanical investigations and clinical studies are necessary to evaluate whether this technique can translate into improved stability and functional outcomes.

## Data Availability

No datasets were generated or analysed during the current study.

## Abbreviations

ACJ: Acromioclavicular joint
CC: Coracoclavicular (Ligament)
APF: Acromial periosteal flap
CC: Coracoclavicular (Ligament)
ACLC: Acromioclavicular ligament complex
